# Modelling the cost-effectiveness of a new infant vaccine to prevent tuberculosis disease in children in South Africa

**DOI:** 10.1186/1478-7547-12-20

**Published:** 2014-09-16

**Authors:** Liezl Channing, Edina Sinanovic

**Affiliations:** 1 Health Economics Unit, School of Public Health and Family Medicine, Faculty of Health Sciences, University of Cape Town, Anzio Road, Observatory, 7925 Cape Town, South Africa

**Keywords:** Cost-effectiveness analysis, New TB vaccine, Markov modelling, Childhood TB, South Africa, Tuberculosis, BCG vaccine

## Abstract

**Background:**

Tuberculosis remains the leading cause of death in South Africa. A number of potential new TB vaccine candidates have been identified and are currently in clinical trials. One such candidate is MVA85A. This study aimed to estimate the cost-effectiveness of adding the MVA85A vaccine as a booster to the BCG vaccine in children from the perspective of the South African government.

**Methods:**

The cost-effectiveness was assessed by employing Decision Analytic Modelling, through the use of a Markov model. The model compared the existing strategy of BCG vaccination to a new strategy in which infants receive BCG and a booster vaccine, MVA85A, at 4 months of age. The costs and outcomes of the two strategies are estimated through modelling the vaccination of a hypothetical cohort of newborns and following them from birth through to 10 years of age, employing 6-monthly cycles.

**Results:**

The results of the cost-effectiveness analysis indicate that the MVA85A strategy is both more costly and more effective – there are fewer TB cases and deaths from TB than BCG alone. The South African government would need to spend an additional USD 1,105 for every additional TB case averted and USD 284,017 for every additional TB death averted. The threshold analysis shows that, if the efficacy of the MVA85A vaccine was 41.3% (instead of the current efficacy of 17.3%), the two strategies would have the same cost but more cases of TB and more deaths from TB would be prevented by adding the MVA85A vaccine to the BCG vaccine. In this case, the government chould consider the MVA85A strategy.

**Conclusions:**

At the current level of efficacy, the MVA85A vaccine is neither effective nor cost-effective and, therefore, not a good use of limited resources. Nevertheless, this study contributes to developing a standardized Markov model, which could be used, in the future, to estimate the potential cost-effectiveness of new TB vaccines compared to the BCG vaccine, in children between the ages of 0–10 years. It also provides an indicative threshold of vaccine efficacy, which could guide future development.

## Background

Tuberculosis (TB) remains the leading cause of death in South Africa with 62,827 deaths (11.6%) in 2010 [1]. South Africa is one of twenty-two high TB burden countries and one of twenty-seven high MDR-TB burden countries. TB/HIV co-infection is also very high, with an estimated 0.5 million new TB cases, of which 65% were in HIV positive individuals [2].

Globally, TB in children remains a neglected priority of stakeholders, including researchers, governments, and policy makers. It is only recently that policy makers and advocates have begun raising the issues around addressing childhood diseases, including child-friendly medicine formulations and diagnostics and ensuring that data relevant to children are available. For this reason, the true extent of the global burden of TB disease in children is not known [3-6], but it is estimated to be around 490,000 cases and 64,000 deaths, annually [2]. Although, proportionally, these numbers are small compared to adolescents and adults, the morbidity and mortality in children tends to be higher as children tend to acquire the more severe forms of TB and treatment may be delayed given the difficulties in diagnosing TB in children. Furthermore, once infected, children become the reservoir for future TB disease [5].

Data on the burden of TB in children in South Africa is inadequate. The limited information available suggests that childhood TB represents about 15-20% of TB disease burden in South Africa [7]. Mortality data shows that TB is the 5^th^ leading cause of death in all children under 14 years, the 6^th^ leading cause in the post-neonatal period (age 29 days to 11 months), and the 4^th^ leading cause in children 1-4 years [1].

The Bacille Calmette-Guérin (BCG) vaccine is, currently, the only commercially available vaccine against tuberculosis. Data on its effectiveness in preventing primary infection and disease progression to pulmonary TB is highly varied – ranging from 0–80%. However, there is consensus that BCG is protective against disseminated forms of TB, including military and meningeal TB [8-12], and a cost-effectiveness analysis, conducted in 2006, declared BCG vaccination to be “highly cost-effective” [13]. South Africa introduced universal BCG vaccination for all infants at birth in 1972 [14].

Modelling studies show that existing strategies alone are not sufficient to achieve the 2050 target of elimination of TB as a public health concern, and that new strategies for prevention (e.g. new tools for diagnosis and new vaccines, particularly new vaccines that both prevent infection and disease progression), and treatment (e.g. shorter, more effective regimens) are needed [15-17].

Following the successful sequencing of the Mycobacterium Tuberculosis (*M.tuberculosis*) genome as well as progress in sequencing BCG, a number of potential new TB vaccine candidates have been identified and are, currently, in Clinical Trials. One such candidate is MVA85A. MVA85A is a “post-exposure” sub-unit vaccine that is designed to boost the immunological response of BCG [18-20].

Resources for TB control are limited and have been further constrained due to the global financial crisis. In addition, a number of new vaccines (e.g. pneumococcal, rotavirus, and human papillomavirus) have been developed over the past decade which increases the competition for these limited resources. Governments and donors need to determine which interventions or set of interventions they should invest in so as to have the greatest impact on their populations’ health.

This study aimed to examine the cost-effectiveness of adding the MVA85A vaccine as a booster to the BCG vaccine in children from the perspective of the South African government.

The economic evaluation was requested by the product developers in 2012 and the study was conceptualized while the Phase IIb clinical trial in infants was still on-going. Despite the disappointing results, which were made available in 2013 [21], we believe that the development of a model, which could be used to assess the potential cost-effectiveness of other new TB vaccines in infants, together with the establishment of a threshold efficacy value contributes to the on-going work in bringing a new TB vaccine to market; consequently, we proceeded with the study and with submission for publication.

## Methods

### Study design

A Markov state transition model was developed in TreeAge Pro Suite® 2012 to reflect the natural course of TB in children. The model compared the existing strategy of BCG vaccination at birth to a new strategy in which infants receive BCG at birth and a booster vaccine, MVA85A, at 4 months of age. The costs and outcomes of the two strategies were estimated through modelling the vaccination of a hypothetical cohort of newborn children and following them from birth through to 10 years of age, employing 6-monthly cycles. A time horizon of 10 years was chosen as this represents the time period over which there is a unique pathway of TB in children. Beyond 10 years, the course of TB tends to mimic that of adults. Furthermore, data on the effectiveness of the MVA85A vaccine in adults is not yet known and data on the effectiveness of the BCG vaccine beyond 10 years is highly varied. Modelling is employed to estimate the cost-effectiveness of a new vaccination strategy as it allows us to extend the costs and outcomes of the two interventions beyond the trial time-horizon of 2 years.

### Model description

**Figure 1:**
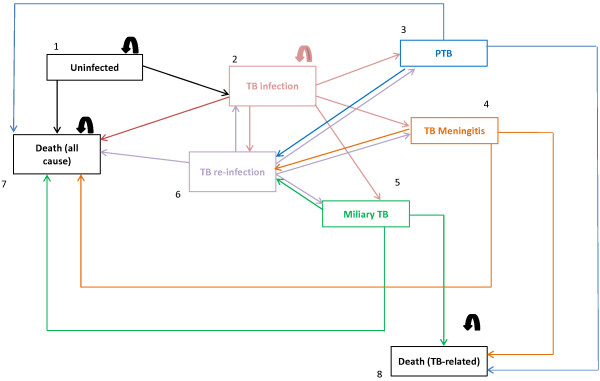
Health states and possible transitions (State diagram).

**Figure 2:**
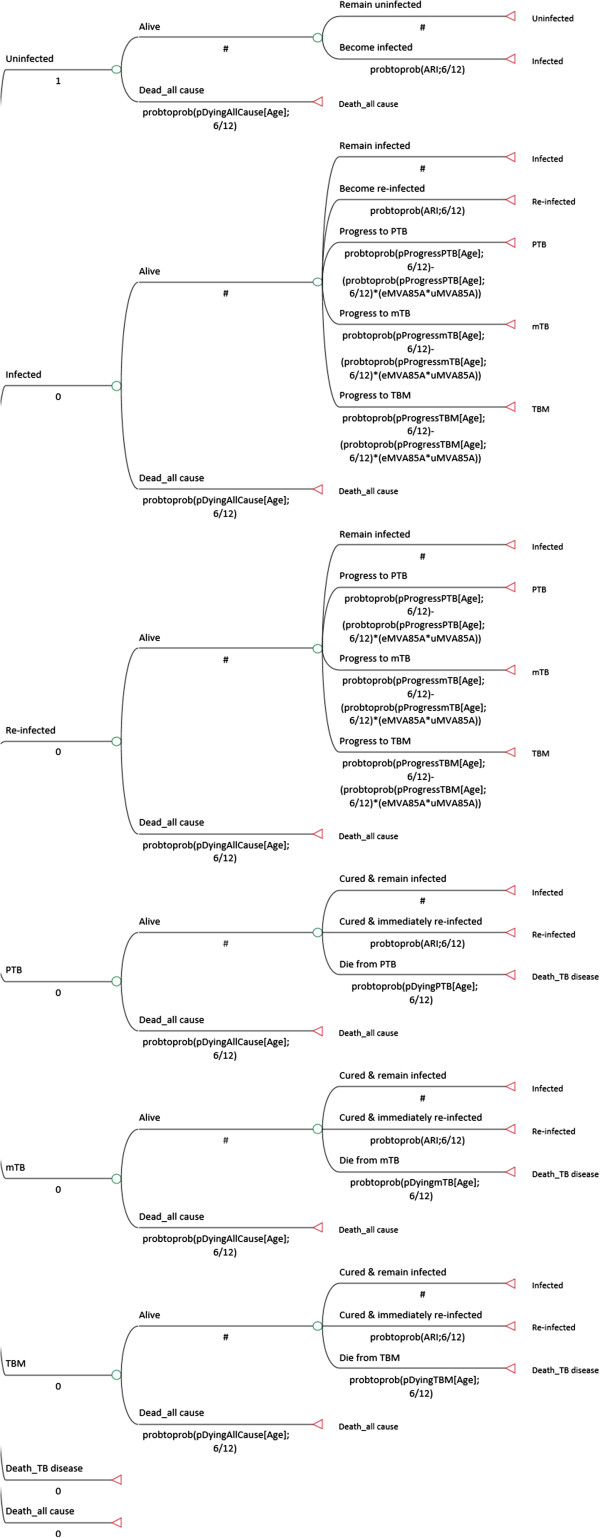
One arm of the Markov model (BCG + MVA85A).

Eight mutually exclusive health (Markov) states representing the natural history of tuberculosis (TB) disease in children were used (Figure 1). Transitions amongst health states were permitted according to specific transition probabilities and specific criteria (Table 1, Figures 1 and 2). Patients could remain in certain health states (e.g. uninfected) and “Death” was considered to be an absorbing state. The model is a static (one Annual Risk of Infection has been used for the entire duration of the model), population-based (a single cohort moves through the model), deterministic (set parameters are used to determine how the cohort moves through the model), closed (new individuals cannot enter the model over the 10-year period), and discrete (events occur at 6-monthly intervals) model.

**Table 1:** Model parameters: estimates of the probability of the events

	**Probability % (range)**	**Reference**
ARI and Annual risk of re-infection	3 (2-4)	[22-25] and expert opinion^a^
Progressing to pulmonary TB		
Age		Assuming an ARI of 3%, calculated using the provincial government Western Cape Department of Health electronic TB database, and expert opinion^a^
0-2	54.19	
3-5	20.37	
6-10	6.60	
Progressing to miliary TB		
Age		
0-2	0.22	
3-5	0.10	
6-10	0.04	
Progressing to TB meningitis		
Age		
0-2	0.52	
3-5	0.14	
6-10	0.10	
Dying from pulmonary TB		
Age		
<3	0.75	
3-5	0.09	
>6	0.59	[26] , the provincial government Western Cape Department of Health electronic TB database, and expert opinion^a^
Dying from miliary TB		
Age		
<3	23.53	
3-5	9.09	
>6	16.66	
Dying from TB meningitis		
Age		
<3	25.00	
3-5	26.66	
>6	20.00	
Dying from other causes		South African 2009 Life Tables [27] and adjusted to remove the risk of dying from TB, and expert opinion^a^
Age		
0-1	0.0429	
1-2	0.0047	
3-4	0.0049	
5	0.0014	
6-10	0.0014	
10	0.0012	
MVA85A efficacy against disease	17.3 (12.3 – 22.3)^b^	
Up-take BCG		[21]
Up-take MVA85A	99.0 (98.5 – 99.5)	[28]
Drop-out rate DTP3 to MCV	85.0 (76.4 – 89.5)	Calculated
Discount rate_outcomes	14.0 (9.5 – 23.1)	[29]
Discount rate_costs	3 (0 – 6)	[30]
	3 (0 – 6)	[30]

^a^Expert opinion provided by Professor Willem Hanekom, Dr Mark Hatherill, Professor Anneke Hesseling, Professor Helen McShane, Dr Hassan Mohammed, Dr Roxana Rustomjee, and Dr Michele Tameris.

^b^In order to assess the sensitivity of the ICER to the vaccine efficacy, we randomly assigned a range of +/-5% to the clinical trial efficacy of 17.3%.

Age-specific risks for progression to three different TB disease states – pulmonary TB (PTB), miliary TB (mTB), and TB meningitis (TBM), and the risk of death from these disease states were reflected in the model together with the risk of TB infection (Table 1). These data have been taken from the published literature, expert opinion, and government data bases such as the South African electronic TB (e-TB) register.

Data on all-cause mortality rates were taken from WHO 2009 Life-Tables [27] and were adjusted to remove the age-specific risk of dying from one of three TB disease states. Once available, data on the efficacy of MVA85A was taken from the results of the Phase IIb Clinical Trial in Worcester, South Africa, which showed the efficacy rate against tuberculosis in infants to be 17.3% [21].

Whilst there is no standardised Markov model for the evaluation of new TB vaccines, other Markov models have been developed and used in childhood vaccines [31-33]. Our model uses the same conceptual framework like these models i.e. it followed a natural history of disease, it included several health states and applied an appropriate time horizon for the study population, and it made use of all the relevant epidemiological parameters, vaccine characteristics and costs. The face validity of our model was established by consulting local experts on assumptions regarding the natural history of childhood TB infection, disease and treatment and resulting model predictions.

Expert opinion was obtained through a meeting of experts in paediatric TB, TB epidemiology, and TB vaccine development. The model and parameters, including assumptions were presented to the experts and their feedback elicited. Follow-up with the experts occurred via e-mail.

The following assumptions were made in our model:

• all children started out uninfected and, once infected, a child could never be uninfected;

• a single Annual Risk of Infection (ARI) of 3% was used throughout the duration of the model, for all age groups, and for both the risk of “TB infection” and “TB reinfection”;

• three age groups (0–2 years, 3–5 years, and 6–10 years) were represented in the model. These represented the ages at which the risks associated with progression to disease and mortality was significantly different;

• the efficacy of BCG vaccine was indirectly included in the model by virtue of the fact that the TB data used in the model has been taken from a setting in which BCG has been routinely administered since the 1970’s [14] and up-take – defined as the proportion of children eligible to receive the vaccine who actually receive the vaccine – is in excess of 95% [28];

• the efficacy of BCG remained constant over the 10-year period and BCG did not have a direct effect on all-cause mortality

• the drop-out rate between the DTP3 vaccine (given at 14 weeks) and the MCV vaccine (given at 9 months) [29] was taken as the proxy for MVA85A vaccine up-take^a^; and,

• vaccine up-take was used together with efficacy to calculate the effectiveness.

The following were excluded from the model: (a) a herd immunity, as humans seem to have a natural resistance to infection with *M.tuberculosis* and to progression to TB disease, which doesn’t appear to be further enhanced by the BCG vaccine [17] and is not being studied for the MVA85A vaccine; (b) isoniazid preventive therapy (IPT), as the effect would have been equal in both arms; (c) BCG disseminated disease (a side-effect of BCG vaccination), as the effect would have been equal in both arms; and, (d) although HIV is an important co-infection in the context of South Africa, it has been excluded from our model as there is no data on the efficacy of MVA85A vaccine given with BCG vaccine in HIV positive infants and, in the Phase IIb study, MVA85A vaccine was not given to infants who had a positive HIV diagnosis by 4 months. In addition, the TB data used will have accounted for the increased risk of TB disease due to HIV status.

### Costing methods

Costs were taken from the perspective of the South African government and were estimated using an ingredients-based costing methodology. All cost data, except for the price of MVA85A, was taken from the raw data collected for a South African study published in 2013; the data were collected in 2009 [34]. The costs were inflated using consumer price index (CPI) figures to 2012 values [35], and then converted to US dollars (USD) at the average exchange rate USD to ZAR for 2012 of USD 1 = ZAR 8.12 [36]. The Oxford Emergent TB Consortium (OETC) provided the price of MVA85A vaccine in USD. All costs were reflected in 2012 USD. The cost for vaccination, diagnosis, and treatment are reflected in Table 2 with details provided in Additional file 1.

**Table 2:** **Cost of vaccination, diagnosis and treatment**^**a **^**in 2012 USD: base-case estimates and source**

**Parameters**	**Value**	**Range**	**References**
Cost of BCG vaccination (USD 2012)^b^	13.57	13.43 – 14.28	[34-36] and personal communication (Arnot, Hayes)^d^
Cost of MVA85A vaccination (USD 2012)^c^	28.22	20.22 – 48.22	[34-36] and personal communication (Oxford Emergent Tuberculosis Consortium (OETC))
Costs of diagnosis & treatment PTB (USD 2012)			[34-36]
0-2 years	406.13		[34-36] and personal communication (Arnot, Hayes, von Zeil)^d^
3-5 years	433.13		
6-10 years	459.29		
Costs of diagnosis & treatment mTB (USD 2012)			
0-2 years	3,184.76		
3-5 years	3,213.57		
6-10 years	3,241.54		
Costs of diagnosis & treatment TBM (USD 2012)			
0-2 years	29,782.98		
3-5 years	29,844.60		
6-10 years	29,881.88		

^a^TB treatment costs: an average weight of 10kg was used for the age group 0-2 years, 20kg for 3-5 years, and 30kg for 6-10 years. As per the South African TB guidelines treatment is given daily (7 days a week) for 6 months (2 months intensive phase and 4 months continuation phase) for pulmonary TB and miliary TB; whereas treatment is given daily for 6-9 months (single phase of treatment) for TB meningitis. Other costs include costs associated with various diagnostics and laboratory monitoring as well as hospital and clinic costs.

^b^The cost of BCG vaccine includes the cost per dose, which includes 40% wastage, the cost for a needle and syringe, and the cost of a clinic visit.

^c^The cost of MVA85A vaccine includes the cost per dose (provided by OETC), the cost for a needle and syringe, and the cost of a clinic visit.

^d^Provided information on South African tender award for TB medicines and BCG vaccine as well as wastage rates for BCG vaccine.

### Effectiveness and cost-effectiveness measurement

As the vaccine was designed to prevent progression from TB infection to TB disease, we calculated the absolute difference in the number of TB cases and TB deaths between the two alternatives. This was achieved through using transition rewards rather than state/incremental rewards. Given that very limited information is available on the utilities associated with the various health states for TB in children, as well as the difficulty in determining these, the Quality-Adjusted Life-Years (QALY) were not used. Likewise, given the controversies around age weighting through welfare interdependence and disability weighting particularly in children, the Disability Adjusted Life-Year (DALY) approach was not considered. The model was designed to determine the number of TB cases averted and the number of TB deaths averted. At the end of the 10-year period the cumulative costs and outcomes of each intervention were used to calculate the cost-effectiveness ratio (CER) (i.e. the cost per TB case averted and the cost per TB death averted) for each intervention. These two cost-effectiveness ratios were compared using an incremental cost-effectiveness ratio (ICER), which represent the additional cost per additional benefit received.

Consistent with recommendations, all future costs and outcomes were discounted at 3% per annum [30].

### Dealing with uncertainty

**Figure 3:**
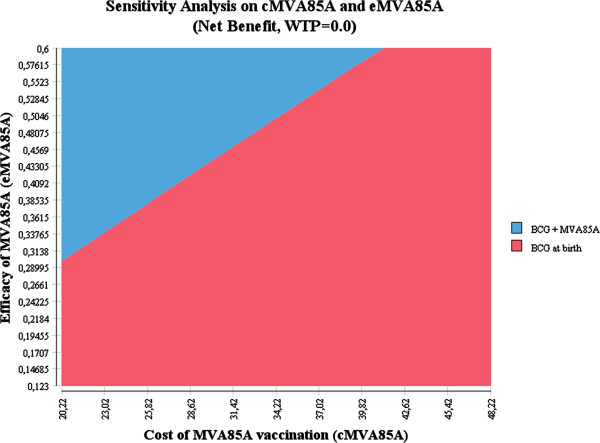
Bivariate analysis of the cost and the efficacy of the MVA85A vaccine.

By its very nature modelling is considered subjective and involves a degree of uncertainty [37]. We, therefore, conducted a univariate sensitivity analysis to check for uncertainty around the discount rate, MVA85A vaccine efficacy, vaccine cost, vaccine coverage, and annual risk of infection. To determine the relationship between the cost and efficacy of the MVA85A vaccine, we conducted a bivariate sensitivity analysis (Figure 3). A threshold analysis was performed to determine the level of efficacy at which the cost of the MVA85A vaccine strategy would equal the cost of the BCG strategy, but produce additional benefits.

## Results

We acknowledge that a cost-effectiveness analysis would not traditionally be carried out on an ineffective intervention. Nevertheless, given that the model was developed while the efficacy study was still on-going, we ran the model with the study outcome of 17.3%. Table 3 shows the discounted and undiscounted 10-year costs, the absolute number of TB cases and TB deaths, and incremental cost-effectiveness ratios (ICERs) associated with adding MVA85A vaccine to the existing strategy of BCG at birth, from the perspective of the South African government. Both the discounted and undiscounted results show that adding the MVA85A vaccine to the BCG vaccine is both more effective and more costly. The base-case scenario reveals ICERs of USD 1,105 per TB case averted and USD 284,017 per TB death averted.

**Table 3:** Cost-effectiveness of adding the MVA85A vaccine to the BCG vaccine, in 2012 USD

**Strategy**	**10-year costs**	**Absolute number of TB cases**	**Absolute number of TB deaths**	**ICER per TB case averted (USD 2012)**	**ICER per TB deaths averted (USD 2012)**
	**(USD 2012)**				
Discounted (3%)					
BCG alone	84.17	0.09101	0.0003501817		
plus MVA85A	98.23	0.07828	0.0003006626	1,105	284,017
Undiscounted					
BCG alone	97.65	0.10627	0.0004174069		
plus MVA85A	109.80	0.09138	0.0003583168	816	205,603
Discounted (6%)					
BCG alone	73.53	0.07885	0.0002969804		
plus MVA85A	89.10	0.06785	0.0002550313	1,416	371,271

### Sensitivity analyses

A summary of the sensitivity analyses for key parameters is provided in Table 4. The results showed that the outcomes were robust; being most sensitive to the ARI, MVA85A vaccine efficacy, and the MVA85A vaccine price. The bivariate analysis shows that the efficacy of the vaccine would need to be significantly greater to justify any increases in cost and that the ICER to sensitive to both cost and efficacy. The threshold analysis shows that at an efficacy of 41.3%, the MVA85A vaccine produces more benefits, but at a cost equal to the BCG vaccine.

**Table 4:** Effect of differing assumptions on the base-case ICER

**Parameter**	**Increase/Decrease in ICER (TB cases averted)**	**Increase/Decrease in ICER (TB deaths averted)**
Annual Risk of Infection (ARI)		
2%	+ 79.86%	+ 79.71%
4%	- 39.88%	- 39.83%
MVA85A vaccine cost (USD)		
20.22	- 48.35%	- 48.35%
48.22	+ 120.87%	+ 120.87%
MVA85A vaccine up-take		
76.4%	+ 0.14%	+ 0.12%
89.5%	- 0.07%	- 0.06%
MVA85A vaccine efficacy		
12.3%	+ 69.90%	+ 69.81%
22.3%	- 38.55%	- 38.52%
Discounting		
0%	-26.15%	- 27.61%
6%	28.14%	+ 30.72%

## Discussion

This study explored the cost-effectiveness of adding the MVA85A vaccine as a booster to the BCG vaccine in children from the perspective of the South African government. The recently published results of the Phase IIb clinical trial conducted in Worcester, South Africa, showed the efficacy of the MVA85A vaccine in preventing TB in infants to be 17.3% [21]. The vaccine can, therefore, be considered ineffective. This has had a noticeable effect on the outcomes of our study. Nevertheless, we proceeded for two reasons – the first being the importance of finalizing the development and testing of a model, which could potentially be used for assessing the cost-effectiveness of other new TB vaccine candidates currently being studied; and the second being the possibility of establishing a threshold value for efficacy which could guide TB vaccine development. To the best of our knowledge, there are no other completed clinical trials or on-going clinical trials evaluating the efficacy of MVA85A vaccine in infants and this is the only published study indicating the efficacy of the vaccine in infants.

At the cost of USD 1,105 per TB case averted and USD 284,017 per TB death averted, adding the MVA85A vaccine as a booster to the BCG vaccine in children not cost-effective when compared to the cost-effectiveness of other childhood vaccines. For instance, a study in Mozambique showed the incremental cost of introducing hepatitis B vaccine into infant immunization services to be USD 1,833 per death averted and USD 47 per DALY averted [38]. A study in Vietnam found routine immunization with rotavirus vaccine to be cost-effective at USD 40 per DALY averted [31]. However, comparison of cost-effectiveness evidence has a number of limitations: different outcome measures, different methodologies, different price levels and different cost base years.

We developed a Markov model which reflects the natural course of TB in children and represents 3 significant age groups associated with progression to TB disease and TB mortality in children up to 10 years. This model could be used to determine the cost-effectiveness of other new TB vaccines being tested in infants and could be modified to different country contexts. In addition, the model could, potentially, be adapted to reflect the different populations that the efficacy of MVA85A continues to be tested in (e.g. HIV-positive adults). Further work would need to be done to calibrate the model.

The limitations of our study arise from the paucity of data on childhood TB during the chemotherapy era, and our inability to access the full South African e-TB register dataset. For these reasons, parameters have been derived from the Western Cape’s e-TB register. As the Western Cape has the third highest number of TB cases in South Africa [39], we do not believe that this has distorted the results. The study assumed an annual risk of infection of 3% and has applied this to the e-TB register data in order to establish the risk of progressing to disease. It was also assumed that the e-TB register data reflected the effectiveness of the BCG vaccine in the population given that the Western Cape has routinely administered BCG since the 1970’s [14] and up-take is in excess of 95% [28].

The threshold analysis shows that, if the efficacy of the MVA85A vaccine was 41.3% (instead of the current efficacy of 17.3%), the two strategies would have the same cost but more cases of TB and more deaths from TB would be prevented by adding the MVA85A vaccine to the BCG vaccine. This result is consistent with a recently published study by Ditkowsky and Swartzman which showed that, at values below 40% efficacy for MVA85A, the MVA85A booster strategy is more expensive and only slightly more effective than BCG alone [40]. They are also similar to earlier analyses of novel vaccines for TB [41,42]. In this case, the South African government could consider the MVA85A strategy, but an affordability study would still need to be conducted.

In addition, sensitisation of the regulatory authority and communities around modestly efficacious vaccines would need to occur to ensure that the new vaccine is licensed, authorised for use, and included in the EPI schedule and that peoples’ confidence in vaccination programmes is not eroded so that there is sufficient up-take of the new vaccine.

## Conclusions

Our findings indicate that, due to its low efficacy, adding MVA85A as a booster to BCG against infant and childhood TB is not cost-effective, and, therefore, not a viable use of limited resources. Nevertheless, our research contributes to developing a standardized Markov model, which could be used, in the future, to estimate the potential cost-effectiveness of new TB vaccines compared to the BCG vaccine, in children between the ages of 0–10 years. It also provides an indicative threshold of vaccine efficacy, which could guide future development.

## Endnote

^a^The second dose of MVA85A vaccine is given at 4-6 months. We, therefore, used the drop-out rate between DTP3 vaccine – given at 3.5 months – and the MCV vaccine – given at 9 months – as the proxy for MVA85A vaccine up-take.

## Abbreviations

ARI: Annual risk of infection; BCG: Bacille Calmette–Guérin; CER: Cost effectiveness ratio; CPI: Consumer price index; DALY: Disability-adjusted life year; DTP3: Diphtheria-tetanus-pertussis; HIV: Human immunodeficiency virus; ICER: Incremental cost effectiveness ratio; mTB: Miliary tuberculosis; MVA85A: Modified Vaccinia Ankara 85A; MVC: Measles-containing vaccine; OETC: Oxford emergent tuberculosis consortium; PTB: Pulmonary tuberculosis; QALY: Quality-adjusted life year; TBM: Tuberculosis meningitis; USD: U.S. Dollars; ZAR: South African Rand.

## Competing interests

The authors declare that they have no competing interests.

## Authors' contributions

LC and ES designed the study, developed the cost-effectiveness model, analysed the data and contributed to the drafting of the manuscript. Both authors read and approved the final manuscript.

## Supplementary Material

Additional file 1 Supplementary information. Click here for file
