# Supplementary material for: Modelling the cost-effectiveness of a new infant vaccine to prevent tuberculosis disease in children in South Africa
Source: Cost Eff Resour Alloc. 2014 Sep 16;12:20. doi: 10.1186/1478-7547-12-20 (PMC4169661; doi:10.1186/1478-7547-12-20)
Supplement: Additional file 1 — Supplementary information. [file 1478-7547-12-20-S1.docx]

**Supplement 1**

Economic data

| CPIX | 2010 | 4.30% | Stats SA 2010 average [29] |
| --- | --- | --- | --- |
|  | 2011 | 5.00% | Stats SA 2011 average [29] |
|  | 2012 | 5.70% | Stats SA 2012 average up to September 2012 [29] |
| USD | 2012 | 8.12 | SA Reserve Bank average 1 Jan 2012 to 31 Oct 2012 [30] |

Cost of vaccination

|  |  |  | **ZAR** | **ZAR** | **ZAR** | **ZAR** | **USD** | **USD** |
| --- | --- | --- | --- | --- | --- | --- | --- | --- |
|  |  |  | **2009** | **2010** | **2011** | **2012** | **2012** | **2012** |
| **BCG** |  | Qty | Unit cost | Unit cost | Unit cost | Unit cost | Unit cost | Total cost |
|  | Clinic visit | 1 | 92.20 | 96.16 | 100.97 | 106.73 | 13.14 | 13.14 |
|  | Needle & Syringe | 1 |  |  |  | 0.63 | 0.08 | 0.08 |
| ZAR 34.32/20 doses | Vaccine | 1 |  |  |  | 1.72 | 0.21 | 0.21 |
|  | Wastage | 0% |  |  |  | 1.72 | 0.21 | 0.21 |
|  |  | 40% |  |  |  | 2.86 | 0.35 | 0.35 |
|  |  | 80% |  |  |  | 8.58 | 1.06 | 1.06 |
|  |  |  | Total cost of vaccination_0% wastage | | | | | 13.43 |
|  |  |  | Total cost of vaccination_40% wastage | | | | | 13.57 |
|  |  |  | Total cost of vaccination_80% wastage | | | | | 14.28 |

|  |  |  | **ZAR** | **ZAR** | **ZAR** | **ZAR** | **USD** | **USD** |
| --- | --- | --- | --- | --- | --- | --- | --- | --- |
|  |  |  | **2009** | **2010** | **2011** | **2012** | **2012** | **2012** |
| **MVA85A** |  | Qty | Unit cost | Unit cost | Unit cost | Unit cost | Unit cost | Total cost |
|  | Clinic visit | 1 | 92.20 | 96.16 | 100.97 | 106.73 | 13.14 | 13.14 |
|  | Needle & Syringe | 1 |  |  |  | 0.63 | 0.08 | 0.08 |
| per dose | Vaccine | 1 |  |  |  |  | 15 | 15 |
|  |  | lower range |  |  |  |  | 7 | 7 |
|  |  | upper range |  |  |  |  | 35 | 35 |
|  |  |  | Total cost of vaccination_USD 15 per dose | | | | | 28.22 |
|  |  |  | Total cost of vaccination_USD 7 per dose | | | | | 20.22 |
|  |  |  | Total cost of vaccination_USD 35 per dose | | | | | 48.22 |

Cost of diagnosis and treatment

|  |  |  | **ZAR** | **ZAR** | **ZAR** | **ZAR** | **USD** | **USD** |
| --- | --- | --- | --- | --- | --- | --- | --- | --- |
|  |  |  | **2009** | **2010** | **2011** | **2012** | **2012** | **2012** |
| **PTB** |  | Qty | Unit cost | Unit cost | Unit cost | Unit cost | Unit cost | Total cost |
|  | CXR | 2 | 220.00 | 229.46 | 240.93 | 254.67 | 31.36 | 62.73 |
|  | TST | 1 | 125.00 | 130.38 | 136.89 | 144.70 | 17.82 | 17.82 |
|  | Culture | 2 | 130.00 | 135.59 | 142.37 | 150.48 | 18.53 | 37.07 |
|  | DST | 0.3 | 235.00 | 245.11 | 257.36 | 272.03 | 33.50 | 10.05 |
|  | Clinic Visit | 7 | 92.20 | 96.16 | 100.97 | 106.73 | 13.14 | 92.01 |
|  | Hospital days | 1 | 1,118.40 | 1,166.49 | 1,224.82 | 1,294.63 | 159.44 | 159.44 |
|  |  |  | Sub-total (excl meds) | | | | | 379.12 |
|  | Medicines | 0-2 years |  |  |  | 219.28 | 27.01 | 406.13 |
|  |  | 3-5 years |  |  |  | 438.55 | 54.01 | 433.13 |
|  |  | 6-10 years |  |  |  | 650.97 | 80.17 | 459.29 |

|  |  |  | **ZAR** | **ZAR** | **ZAR** | **ZAR** | **USD** | **USD** |
| --- | --- | --- | --- | --- | --- | --- | --- | --- |
|  |  |  | **2009** | **2010** | **2011** | **2012** | **2012** | **2012** |
| **mTB** |  | Qty | Unit cost | Unit cost | Unit cost | Unit cost | Unit cost | Total cost |
|  | CXR | 2 | 220.00 | 229.46 | 240.93 | 254.67 | 31.36 | 62.73 |
|  | TST | 1 | 125.00 | 130.38 | 136.89 | 144.70 | 17.82 | 17.82 |
|  | Culture | 2 | 130.00 | 135.59 | 142.37 | 150.48 | 18.53 | 37.07 |
|  | DST | 0.3 | 235.00 | 245.11 | 257.36 | 272.03 | 33.50 | 10.05 |
|  | Clinic Visit | 8 | 92.20 | 96.16 | 100.97 | 106.73 | 13.14 | 105.15 |
|  | Hospital days | 15 | 1,118.40 | 1,166.49 | 1,224.82 | 1,294.63 | 159.44 | 2,391.65 |
|  | Out-pt visit days | 10 | 372.80 | 388.83 | 408.27 | 431.54 | 53.15 | 531.48 |
|  |  |  | Sub-total (excl meds) | | | | | 3,155.95 |
|  | Medicines | 0-2 years |  |  |  | 233.94 | 28.81 | 3,184.76 |
|  |  | 3-5 years |  |  |  | 467.88 | 57.62 | 3,213.57 |
|  |  | 6-10 years |  |  |  | 694.95 | 85.59 | 3,241.54 |

|  |  |  | **ZAR** | **ZAR** | **ZAR** | **ZAR** | **USD** | **USD** |
| --- | --- | --- | --- | --- | --- | --- | --- | --- |
|  |  |  | **2009** | **2010** | **2011** | **2012** | **2012** | **2012** |
| **MTB** |  | Qty | Unit cost | Unit cost | Unit cost | Unit cost | Unit cost | Total cost |
|  | CXR | 2 | 220.00 | 229.46 | 240.93 | 254.67 | 31.36 | 62.73 |
|  | TST | 2 | 125.00 | 130.38 | 136.89 | 144.70 | 17.82 | 35.64 |
|  | Culture | 0.3 | 130.00 | 135.59 | 142.37 | 150.48 | 18.53 | 5.56 |
|  | DST | 2 | 235.00 | 245.11 | 257.36 | 272.03 | 33.50 | 67.00 |
|  | Clinic visit | 2 | 92.20 | 96.16 | 100.97 | 106.73 | 13.14 | 26.29 |
|  | Hospital days | 180 | 1,118.40 | 1,166.49 | 1,224.82 | 1,294.63 | 159.44 | 28,699.78 |
|  | Out-pt visit days | 10 | 372.80 | 388.83 | 408.27 | 431.54 | 53.15 | 531.48 |
|  | CT scan | 1 | 1,522.00 | 1,587.45 | 1,666.82 | 1,761.83 | 216.98 | 216.98 |
|  | CSF | 1 | 185.00 | 192.96 | 202.60 | 214.15 | 26.37 | 26.37 |
|  |  |  | Sub-total (excl meds) | | | | | 29,671.84 |
|  | Medicines | 0-2 years |  |  |  | 902.47 | 111.15 | 29,782.98 |
|  |  | 3-5 years |  |  |  | 1402.77 | 172.76 | 29,844.60 |
|  |  | 6-10 years |  |  |  | 1705.44 | 210.04 | 29,881.88 |

Medicine costs

| **PTB** |  |  |  |  |  |  |  |
| --- | --- | --- | --- | --- | --- | --- | --- |
|  |  |  |  |  | **ZAR** | **ZAR** | **ZAR** |
|  |  | No. Of tabs | Duration | Total tabs | Cost/tab | Total cost | TOTAL |
| Based on 10kg child (0-2 years) |  |  |  |  |  |  |  |
| Intensive | RH 60/60 | 2 | 61 | 122 | 0.54 | 66.23 |  |
|  | Z 500 | 0.75 | 61 | 46 | 0.45 | 20.59 |  |
| Continuation | RH 60/60 | 2 | 122 | 244 | 0.54 | 132.46 | 219.28 |
|  |  |  |  |  |  |  |  |
| Based on 20kg child (3-5 years) |  |  |  |  |  |  |  |
| Intensive | RH 60/60 | 4 | 61 | 244 | 0.54 | 132.46 |  |
|  | Z 500 | 1.5 | 61 | 92 | 0.45 | 41.18 |  |
| Continuation | RH 60/60 | 4 | 122 | 488 | 0.54 | 264.91 | 438.55 |
|  |  |  |  |  |  |  |  |
| Based on 30kg child (6-10 years) |  |  |  |  |  |  |  |
| Intensive | RH 60/60 | 6 | 61 | 366 | 0.54 | 198.69 |  |
|  | Z 500 | 2 | 61 | 122 | 0.45 | 54.91 |  |
| Continuation | RH 60/60 | 6 | 122 | 732 | 0.54 | 397.37 | 650.97 |

| **mTB** |  |  |  |  |  |  |  |
| --- | --- | --- | --- | --- | --- | --- | --- |
|  |  |  |  |  | **ZAR** | **ZAR** | **ZAR** |
|  |  | No. Of tabs | Duration | Total tabs | Cost/tab | Total cost | TOTAL |
| Based on 10kg child (0-2 years) |  |  |  |  |  |  |  |
| Intensive | RH 60/60 | 2 | 61 | 122 | 0.54 | 66.23 |  |
|  | Z 500 | 0.75 | 61 | 46 | 0.45 | 20.59 |  |
|  | E 400 | 0.5 | 61 | 31 | 0.48 | 14.66 |  |
| Continuation | RH 60/60 | 2 | 122 | 244 | 0.54 | 132.46 | 233.94 |
|  |  |  |  |  |  |  |  |
| Based on 20kg child (3-5 years) |  |  |  |  |  |  |  |
| Intensive | RH 60/60 | 4 | 61 | 244 | 0.54 | 132.46 |  |
|  | Z 500 | 1.5 | 61 | 92 | 0.45 | 41.18 |  |
|  | E 400 | 1 | 61 | 61 | 0.48 | 29.32 |  |
| Continuation | RH 60/60 | 4 | 122 | 488 | 0.54 | 264.91 | 467.88 |
|  |  |  |  |  |  |  |  |
| Based on 30kg child (6-10 years) |  |  |  |  |  |  |  |
| Intensive | RH 60/60 | 6 | 61 | 366 | 0.54 | 198.69 |  |
|  | Z 500 | 2 | 61 | 122 | 0.45 | 54.91 |  |
|  | E 400 | 1.5 | 61 | 92 | 0.48 | 43.99 |  |
| Continuation | RH 60/60 | 6 | 122 | 732 | 0.54 | 397.37 | 694.95 |

| **TBM** |  |  |  |  |  |  |  |
| --- | --- | --- | --- | --- | --- | --- | --- |
|  |  |  |  |  | ZAR | ZAR | ZAR |
|  |  | No. Of tabs | Duration | Total tabs | Cost/tab | Total cost | TOTAL |
| Based on 10kg child (0-2 years) |  |  |  |  |  |  |  |
| Single phase of treatment | RH 60/60 | 4 | 229 | 916 | 0.54 | 497.26 |  |
|  | Z 500 | 0.75 | 229 | 172 | 0.45 | 77.30 |  |
|  | Eto 250 | 0.75 | 299 | 224 | 1.46 | 327.91 | 902.47 |
|  |  |  |  |  |  |  |  |
| Based on 20kg child (3-5 years) |  |  |  |  |  |  |  |
| Single phase of treatment | RH 60/60 | 6 | 229 | 1374 | 0.54 | 745.89 |  |
|  | Z 500 | 1.5 | 229 | 344 | 0.45 | 154.60 |  |
|  | Eto 250 | 1.5 | 229 | 344 | 1.46 | 502.28 | 1402.77 |
|  |  |  |  |  |  |  |  |
| Based on 30kg child (6-10 years) |  |  |  |  |  |  |  |
| Single phase of treatment | RH 60/60 | 6 | 229 | 1374 | 0.54 | 745.89 |  |
|  | Z 500 | 2 | 229 | 458 | 0.45 | 206.14 |  |
|  | Eto 250 | 2.25 | 229 | 515 | 1.46 | 753.42 | 1705.44 |
